# Minor immunomodulatory effects of psychotropics suggested in severe mental disorders: Associations of antipsychotics with beta defensin 2, antidepressants with C-reactive protein, and mood stabilizers with soluble interleukin 2 receptor

**DOI:** 10.1192/j.eurpsy.2025.10104

**Published:** 2025-09-12

**Authors:** Monica B. E. G. Ormerod, Mashhood A. Sheikh, Thor Ueland, Gabriela Hjell, Linn Rødevand, Linn Sofie Sæther, Synve Hoffart Lunding, Ingrid Torp Johansen, Dimitrios Andreou, Torill Ueland, Trine Vik Lagerberg, Ingrid Melle, Srdjan Djurovic, Ole A. Andreassen, Nils Eiel Steen

**Affiliations:** 1 Division of Mental Health and Addiction, Oslo University Hospital, Oslo, Norway; 2 Institute of Clinical Medicine, University of Oslo, Oslo, Norway; 3 Division of Surgery, Inflammatory Medicine and Transplantation, Oslo University Hospital, Oslo, Norway; 4 Division of Internal Medicine, University Hospital of North Norway, Tromsø, Norway; 5 Department of Psychiatry, Østfold Hospital, Graalum, Norway; 6 Department of Psychology, University of Oslo, Oslo, Norway; 7 Department of Clinical Neuroscience, Centre for Psychiatry Research, Karolinska Institutet & Stockholm Health Care Services, Stockholm Region, Stockholm, Sweden; 8 Division of Mental Health and Substance Abuse, Diakonhjemmet Hospital, Oslo, Norway; 9 Department of Medical Genetics, Division of Laboratory Medicine, Oslo University Hospital and University of Oslo, Oslo, Norway; 10 Department of Clinical Science, University of Bergen, Bergen, Norway

**Keywords:** beta defensin 2, bipolar disorder, C-reactive protein, immunomodulation, schizophrenia, soluble interleukin 2 receptor

## Abstract

**Background:**

Immunomodulatory effects of psychotropic agents used to treat severe mental disorders (SMDs) have been suggested. We investigated associations between immune marker levels and antipsychotic- (AP), antidepressant- (AD), and mood stabilizing agents (MS) use in SMDs.

**Methods:**

We included 1215 participants with SMDs (777 with schizophrenia spectrum disorders and 438 with bipolar disorders). Circulating levels of 45 immune markers were determined by enzyme-immunoassay or immunoturbidimetry and analyzed for associations with use, doses, and serum concentrations of AP, AD, and MS. Extensive adjustments for potential confounders were performed. Immune marker levels of 1008 healthy controls served as a reference.

**Results:**

AP use was significantly associated with higher plasma levels of beta defensin 2 (BD-2) (*β* = 0.094, *p* = 0.8E-4), AD use with higher serum levels of CRP (*β* = 0.072, *p* = 0.8E-3), and MS use with higher plasma levels of soluble interleukin 2 receptor (sIL-2R) (*β* = 0.063, *p* = 0.9E-4). These findings were paralleled by positive associations with psychotropic agent dose and serum concentrations: AP dose was associated with BD-2 levels (*β* = 0.045, *p* = 2.3E-4), AD dose with CRP levels (*β* = 0.039, *p* = 0.001), MS dose with sIL-2R levels (*β* = 0.048, *p* = 0.001), and serum concentration of AD was nominally positively associated with CRP (*β* = 0.072, *p* = 0.002).

**Conclusions:**

The findings suggest that AP and MS use affect pathways involved in immune homeostasis and inflammatory regulation in individuals with SMDs, while AD use augments low-grade systemic inflammation reflected by CRP.

## Graphical abstract

**Figure:**
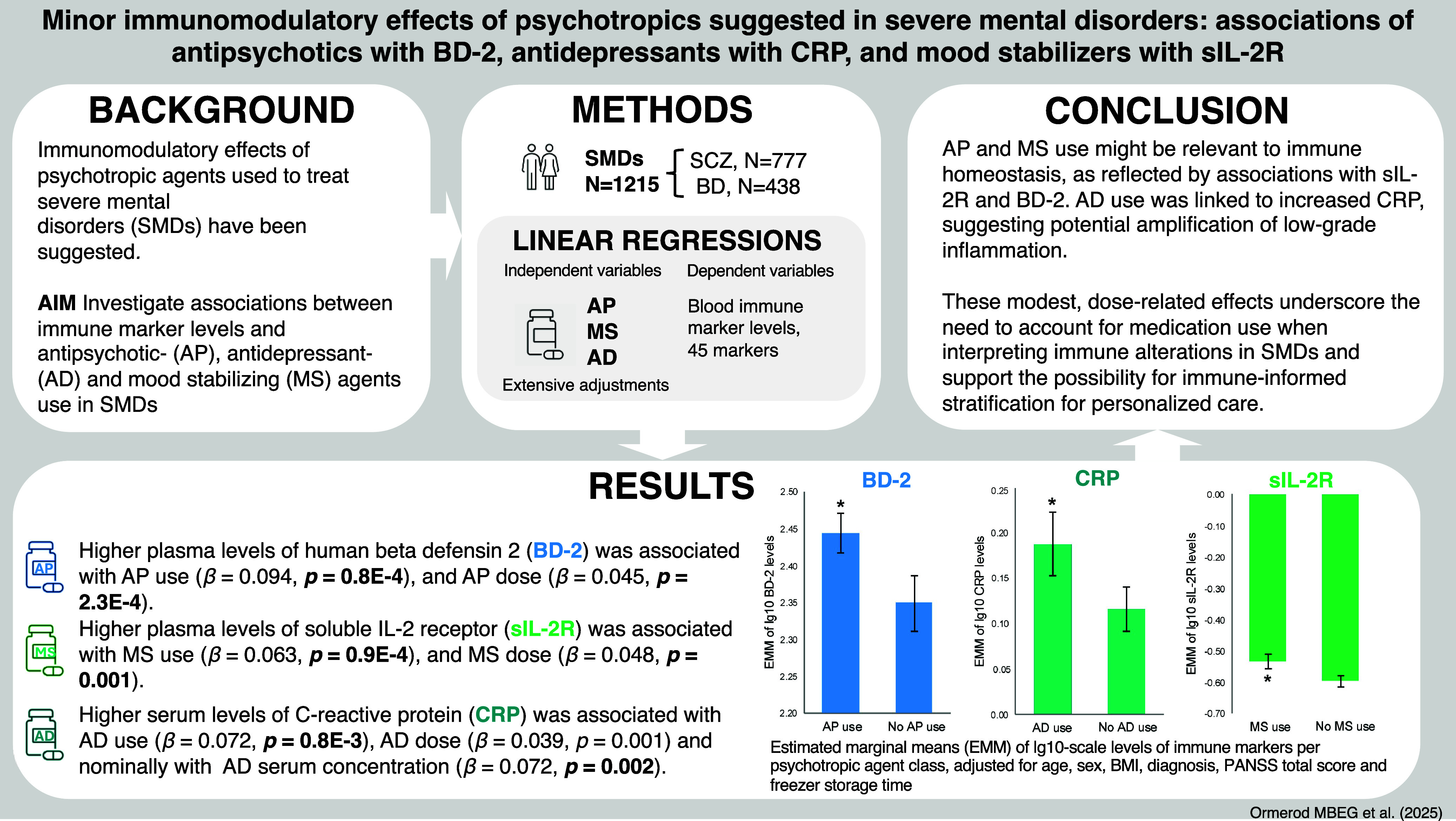

## Introduction

Severe mental disorders (SMDs), including schizophrenia spectrum disorders (SCZ) and bipolar disorder (BD), share etiopathogenic factors and clinical characteristics such as psychotic symptoms, cognitive impairment, and mood dysregulation [1]. Their pharmacological treatment also overlaps considerably, with antipsychotic- (AP), antidepressant- (AD), and mood stabilizing agents [MS; antiepileptics (AE) and lithium] prescribed across diagnoses [2–4]. While modulation of neurotransmitters is a major target for SMDs pharmacotherapy [5, 6], the mechanisms behind treatment effects are not completely understood. One area of growing interest is their potential immunomodulatory effects, given the increasing evidence implicating the immune system in SMDs.

Involvement of immune and inflammatory pathways in SMDs pathophysiology is indicated by a large volume of data, that is associations with infections and autoimmune diseases [7, 8], alterations in peripheral and central immune factors [9–11], and links to immune-related genetic loci [12–14]. However, the extent to which psychotropic agents impact the altered immune activity and inflammation remains unclear.

While the use of AP, AD, and MS has been linked to immunomodulatory effects [15–32], small sample sizes and significant heterogeneity of study designs and findings indicate that additional studies are required [33, 34]. A recent meta-analysis based on individuals with first-episode psychosis suggests anti-inflammatory properties of AP by modulating both pro- and anti-inflammatory cytokines, including interleukin (IL)-1β, IL-4, IL-6, IL-10, interferon (IFN)-γ, and tumor necrosis factor (TNF)-α [15, 22, 24, 33, 34]. Although based on longitudinal data, the individual sample sizes were generally small with heterogenous treatment regimens. Similarly, immunomodulatory effects have been indicated for AD; however, the evidence is conflicting and primarily based on studies in major depressive disorder (MDD) [20, 21, 34–37]. A recent review reported that associations between use of various ADs and levels of IFN-γ, C-reactive protein (CRP), IL-1β, IL-1α, IL-6, and TNF-α varied from positive to negative and non-significant in MDD [36]. To the best of our knowledge, there are only a couple of studies of AD use and immune markers in SCZ [38] and BD [39], reporting negative associations between escitalopram use and levels of CRP and IL-6, and no associations between escitalopram use on CRP levels, respectively. For MS, a systematic review found that lithium use in BD was associated with increased levels of IL-4 and TNF-α, whilst no associations were found with levels of IL-1β, IL-6, and IL-8 [26]. The number of studies was insufficient to draw inferences regarding AE [26, 32]. A more recent study indicated reduced levels of IL-1β, MIF, and IL-6 in a BD group treated with lamotrigine compared to a control BD group treated with sodium valproate [40]. However, existing studies are often limited by small or modest sample sizes, focus on individual agents, and lack of dose or serum concentration data.

To address these limitations, we aimed to clarify associations between use of psychotropic agent classes and immune marker levels in SMDs by using a large sample, and comprehensive assessment of immune markers, including dose and serum analyses, and making extensive covariate adjustments. We adopted a novel approach by grouping patients according to the medication class used. This may yield more robust results and reflects the perspective that clinicians, in large part, relate to medication classes regarding treatment efficacy in SMDs. We hypothesized that use of AP, MS, and AD would be associated with changes in immune marker levels, indicating a reduction in immune and inflammatory dysregulation. Immune markers of healthy controls (HC) were included as a reference.

## Methods

### Study setting

The sample comprises patients and HC from the Thematically Organized Psychosis (TOP) study in Oslo, Norway. Patients are continuously recruited from major hospitals in Oslo if they meet the criteria for SCZ and BD according to the Diagnostic and Statistical Manual of Mental Disorders (DSM)-IV [41]. HC are randomly selected from the population registry data within the same catchment area. General inclusion criteria are age 18–65 years and sufficient Scandinavian language skills to complete the study. Exclusion criteria include severe neurological or somatic illness affecting brain functioning, IQ < 70, or a history of severe head trauma. For HC, additional exclusion criteria are substance abuse or dependency, and SMDs in close relatives. Recruitment for this study took place between 2002 and 2018.

### Sample

The sample comprises 2223 TOP study participants with available immune marker data: 777 with SCZ (schizophrenia: 443; schizophreniform disorder: 44; schizoaffective disorder: 111; and psychotic disorder not otherwise specified: 179), 438 with BD (bipolar I disorder: 271; bipolar II disorder: 143; and bipolar disorder not otherwise specified: 24), and 1008 HC. Participants with CRP levels >10.0 were excluded to minimize the influence of acute infection on immune marker levels.

### Clinical assessments

Patient interviews were performed by trained clinical psychologists and medical doctors, obtaining sociodemographic, psychiatric, and somatic data. Diagnoses were established using the Structured Clinical Interview for DSM-IV Axis I Disorders, including modules for SMDs and substance use disorders [42]. Inter-rater reliability in the TOP study has shown kappa values of 0.92–0.99 [43]. Symptom severity was evaluated with the Positive and Negative Syndrome Scale (PANSS) [44]. Somatic examinations, including height and weight for body mass index (BMI) calculations, were also performed.

### Psychotropic agents

Information on psychotropic agent use and dose at inclusion was collected through interviews and medical records. Psychotropic agent classes “AP use,” “AD use,” and “MS use (AE and lithium)” were dichotomized as “use” (participants using one or more agents within the class) versus “no use.” Patient participants could be included in multiple classes. Defined daily doses (DDD; WHO, https://www.whocc.no/atc_ddd_index/) were summed within each psychotropic agent class (“AP DDD,” “AD DDD,” and “MS DDD”).

Serum concentrations were measured from fasting blood samples, with a mean sampling time of 09:42 (median 09:30, range 07:30–15:15), drawn from the antecubital vein into serum vials without gel within two weeks of symptom assessment. Samples (*≥* 0.5 mL) were centrifuged, stored at 4 °C, and transported to the laboratory at the Department of Clinical Pharmacology, St. Olav University Hospital, Norway, within the next working day [see [45–47]. Relative serum concentrations were calculated by dividing each serum concentration by the midpoint of its reference interval [48]; these were then summed within each class to yield total serum concentration. Details on psychotropic agent use and concentrations are presented in Table 1 and Supplementary Table 1. Information on somatic agent use, including anti-inflammatory and cardiometabolic agents, is provided in Supplementary Table 2.

**Table 1.** Sample descriptives

	SCZ (N = 777)		BD (N = 438)		HC (N = 1008)		*p*-value	Group comparisons
	N	M (IQR) or %	N	M (IQR) or %	N	M (IQR) or %		
Age (years)	777	27.0 (14)	438	30.0 (17)	1007	32.0 (14)	**<0.001**	SCZ < BD, HC
Sex (males)	464	59.7	117	40.4	543	53.9	**<0.001**	BD < SCZ, HC
BMI (kg/m^2^)	728	25.1 (6.6)	421	25.4 (5.5)	947	24.2 (4.4)	**<0.001**	HC < SCZ, BD
CRP (mg/L)	720	1.20 (2.10)	420	1.00 (1.78)	961	0.90 (1.20)	**<0.001**	HC < SCZ, BD
Freezer storage time (years)	773	9 (6)	436	8 (7)	1006	6 (7)	**<0.001**	HC < SCZ, BD
PANSS total score	762	61.0 (20)	432	44.0 (12)	-	-	**<0.001**	BD < SCZ
Smoking (yes)	413	53.2	222	50.7	-	-	**<0.001**	BD < SCZ
Education (years)	764	13 (4)	432	15 (4.75)	-	-	**<0.001**	SCZ < BD
Duration of illness (years)	769	4 (8)	435	9 (13)	-	-	**<0.001**	SCZ < BD
Comorbid substance use disorder (yes)	187	24.1	95	21.7	-	-	0.346	-
Illicit substance use (past 2 weeks) (yes)	30	3.9	30	6.8	-	-	**0.021**	SCZ < BD
*Psychotropic agents*								
AP^a^ use (yes)	648	83.4	212	48.4	-	-	**<0.001**	BD < SCZ
One AP	648	83.4	212	48.4	-	-	**<0.001**	BD < SCZ
Two APs	170	21.9	23	5.3	-	-	**<0.001**	BD < SCZ
Three APs	20	2.6	2	0.5	-	-	**0.008**	BD < SCZ
No AP	129	16.6	226	51.6	-	-	**<0.001**	SCZ < BD
AP DDD	619	0.80 (1.50)	211	0.00 (0.62)	-	-	**<0.001**	BD < SCZ
AP serum concentration (nmol/L)^b^	682	0.44 (0.85)	397	0.0 (0.0)	-	-	**<0.001**	BD < SCZ
AD^c^ use (yes)	220	28.3	156	35.6	-	-	**0.008**	SCZ < BD
One AD	220	28.3	156	35.6	-	-	**0.008**	SCZ < BD
Two ADs	18	2.3	14	3.2	-	-	0.358	-
No AD	557	71.6	282	64.4	-	-	**0.008**	BD < SCZ
AD DDD	205	0.00 (0.5)	136	0.00 (1.0)	-	-	0.144	-
AD serum concentration (nmol/L)^b^	742	0.0 (0.0)	411	0.0 (0.0)	-	-	**0.027**	SCZ < BD
MS use (yes)	108	13.9	232	53.0	-	-	**<0.001**	SCZ < BD
One AE^d^	95	12.2	166	37.9	-	-	**<0.001**	SCZ < BD
Two AEs^d^	4	0.5	9	2.1	-	-	**0.012**	SCZ < BD
Three AEs^d^	1	0.1	1	0.2	-	-	0.681	-
No AE^d^	682	87.7	272	62.1	-	-	**<0.001**	BD < SCZ
Lithium	18	2.3	82	18.7	-	-	**<0.001**	SCZ < BD
No MS	669	86.1	206	47.0	-	-	**<0.001**	BD < SCZ
MS DDD	108	0.0 (0.0)	232	0.08 (1.0)	-	-	**<0.001**	SCZ < BD
DDD AE	95	0.0 (0.0)	166	0.0 (0.6)	-	-	**<0.001**	SCZ < BD
DDD lithium	18	0.0 (0.0)	82	0.0 (0.0)	-	-	**<0.001**	SCZ < BD
MS serum concentration (nmol/L)^b^	736	0.0 (0.0)	338	0.0 (0.0)	-	-	**<0.001**	SCZ < BD
AE serum concentration (nmol/L)^b^	724	0.0 (0.0)	280	0.0 (0.0)	-	-	**<0.001**	SCZ < BD
Lithium serum concentration (nmol/L)^b^	16	0.0 (0.0)	71	0.0 (0.0)	-	-	0.303	-
Use of anxiolytics/sedatives/hypnotics^e^	80	10.3	43	9.8	-	-	0.791	-
Use of stimulants^f^	8	1.0	6	1.4	-	-	0.594	-

Abbreviations: AD, antidepressant agent; AD, antiepileptic agent; AP, antipsychotic agent; BD, bipolar disorders; BMI, body mass index; DDD, defined daily dose; HC, healthy controls; IQR, interquartile range; M, median; MS, mood stabilizing agent; PANSS, Positive and Negative Syndrome Scale; SCZ, schizophrenia spectrum disorders; no AP/AD/MS, using no agent; one AP/AD/MS, using at least one agent; two AP/AD/MS, using at least two agents; Three AP/AD/MS, using at least three agents.

*Note*: Patients using psychotropic agents [median (IQR)], a) DDD and b) serum concentrations: SCZ: a) AP 1.00 (1.00), AD 1.00 (1.00), MS 0.67 (0.69), and b) AP 0.56 (0.70), AD 0.34 (0.51), MS 0.49 (0.50); BD: a) AP 0.67 (0.67), AD 1.00 (1.25), MS 1.00 (0.83), and b) AP 0.30 (0.40), AD 0.32 (0.52), MS 0.66 (0.38). Group comparisons a) AP *p* < 0.001 (BD < SCZ), AD *p* = 0.766, MS *p* = 0.020 (SCZ < BD), and b) AP *p* < 0.001 (BD < SCZ), AD *p* = 0.032 (BD < SCZ), MS *p* < 0.001 (SCZ < BD).

a AP: amisulpride, aripiprazole, dehydroaripiprazole, chlorpromazine, chlorprothixene, clozapine, flupentixol, haloperidol, levomepromazine, olanzapine, paliperidone, perphenazine, quetiapine, risperidone, ziprasidone, zuclopenthixol.

b Serum concentration AP, AD, AE, MS, lithium: the sum of relative serum concentrations [serum concentrations divided by the middle values of their reference intervals (Heimke et al. 2018)] within the psychotropic agent classes. Serum concentrations with M (IQR) given by [0.0 (0.0)], reflect that a large portion of the participants had no measurable serum concentrations of the respective agent classes. Serum concentration data and levels are based on laboratory measurements in combination with documented use or non-use of the agent.

c AD: amitriptyline, bupropion, citalopram, clomipramine, duloxetine, escitalopram, fluoxetine, mianserin, mirtazapine, paroxetine, reboxetine, sertraline, trimipramine, venlafaxine.

d AE: carbamazepine, clonazepam, gabapentin, lamotrigine, pregabalin, topiramate, valproate.

e Alimemazine, buspirone, diazepam, flunitrazepam, hydroxyzine, melatonin, nitrazepam, oxazepam, zolpidem, zopiclone.

f Atomoxetine, dexamphetamine, metylphenidate.

### Immune markers

Blood samples for analysis of immune markers (Table 2, Supplementary Table 3) were drawn within two weeks of symptom severity assessment on EDTA vials. Plasma was isolated within the next working day and stored at −80 °C. For HC, the mean sampling time was 12:31 (median 11:20, range 07:20–19:00); in patients, sampling coincided with that for serum concentration analyses [49, 50]. Serum CRP was analyzed by immunoturbidimetry at the Department of Medical Biochemistry, Oslo University Hospital, Norway. The remaining immune markers were analyzed in duplicate using enzyme immunoassays (EIA) with R&D Systems antibodies (Minneapolis, MN, USA) in 384-well format, performed at the Research Institute of Internal Medicine, Oslo University Hospital. Assays were conducted using a Selma pipetting robot and a Biotek dispenser/washer. Absorbance was measured at 450 nm (with 540 nm correction) using a BIO-RAD (Hercules, CA, USA) ELISA plate reader. All EIAs had inter- and intra-assay coefficients of variation <10%. Previous publications have reported on immune marker variations and associations with psychotropic agent use in the TOP sample [e.g. [49, 51–56]], including diurnal and postprandial variations [53, 57–59]. All blood sampling was performed by a single laboratory.

**Table 2.** List of 45 immune markers assessed

Group	Name
*Interleukins and related markers*	
*IL–1 system*	Interleukin 1 receptor antagonist A (IL–1RA)
	Interleukin 18 (IL–18)
	Interleukin 18 binding protein (IL–18BP)
	Interleukin 18 receptor accessory protein (IL–18RAP)
	Interleukin 18 receptor 1 (IL–18R1)
*TNF system*	Soluble TNF receptor 1 (sTNF-R1)
	B-cell activating factor (BAFF)
	Osteoprotegerin (OPG)
	A proliferation-inducing ligand (APRIL)
*IL–6 family*	Soluble glycoprotein 130 (gp130)
*Chemokines*	Growth-regulated oncogene alpha (GROα/CXCL1)
	Stromal cell-derived factor 1 alpha (SDF1α/CXCL12)
	Eotaxin (CCL11)
	Regulated upon activation normal T-cell expressed and secreted (RANTES/CCL5)
	CXC motif chemokine ligand 16 (CXCL16)
*Cell adhesion molecules (CAMs)*	Mucosal vascular addressin cell adhesion molecule–1 (MAdCAM–1)
	Junctional adhesion molecule-A (JAMA)
	Neural cadherin (NCAD)
	Intercellular adhesion molecule–1 (ICAM–1)
	Vascular cell adhesion molecule–1 (VCAM–1)
	P-selectin (PSEL)
	Activated leukocyte cell adhesion molecule (ALCAM)
*Defensins*	Human neutrophil peptides 1–3 (HNP1–3)
	Human beta defensin 1 (BD–1)
	Human beta defensin 2 (BD–2)
*Leukocyte activation*	Soluble interleukin 2 receptor (sIL–2R)
	Macrophage migration inhibitory factor (MIF)
	Soluble cluster of differentiation 14 (sCD14)
*Blood–brain barrier (BBB) integrity*	S100 calcium-binding protein B (S100B)
	Furin
	Glial fibrillary acidic protein (GFAP)
	Neuron-specific enolase (NSE/ENO2)
*Acute phase proteins*	Alpha–2-macroglobulin (A2M)
	C-reactive protein (CRP)
*Extracellular matrix-related markers*	Cathepsin S (CAT-S)
	Galectin–3 (Gal–3)
	Serpin family A member 3 (SERPINA3)
	Chitinase 40 (YKL–40)
*Oxidative stress*	Myeloperoxidase (MPO)
	Parkinsons disease protein 7/protein deglycase DJ–1 (PARK7/DJ–1)
*Vascular inflammation*	Long pentraxin–3 (PTX3)
	Von Willebrand factor (vWF)
	Insulin-like growth factor binding protein 4 (IGFBP4)
*Others*	Brain-derived neurotrophic factor (BDNF)
	Dickkopf–1 (DKK1)

### Statistical analysis

Statistical analyses were conducted using SPSS for Windows, version 29 (SPSS Inc., Chicago, IL, USA). Normality was assessed by inspecting histograms, Q–Q-plots, and Kolmogorov–Smirnov statistics. Sample descriptives and immune marker data were analyzed using chi-square tests for categorical variables and Kruskal-Wallis and Mann–Whitney *U* tests for continuous variables. Immune marker data were log10-transformed for use in linear regressions, and outliers were removed using the interquartile range method [60] (Supplementary Table 4).

Linear regressions were used to separately examine associations between pairs of immune markers (dependent variable) and AP, AD, and MS variables (‘use’ versus ‘no use’; independent variable). Main models were adjusted for age, sex, and BMI [61], diagnosis, PANSS total score [62], and plasma freezer storage time [63]. A significance threshold of *p* < 0.001 was applied to address multiple testing concerns, to accommodate the need to minimize false positives while avoiding an excessive risk of rejecting potentially interesting associations [64, 65]. Sensitivity analyses for significant associations included the following additional covariates: education (years) [61], current smoking (yes/no) [66], duration of illness (years) [67], illicit substance use within the past two weeks (yes/no) [68], time of blood sampling, CRP (except in CRP models), and DDD and serum concentrations, respectively, of medication classes not specifically tested. Secondary linear regressions were conducted for identified pairs of psychotropic agent classes and immune markers, by substituting psychotropic agent class use with DDD or serum concentration, using the same covariates as the main analyses, and adding an adjustment for blood sampling time in the serum concentration analyses. Subgroup analyses of MS use were performed for AE without lithium and lithium without AE. Lastly, the following interactions were explored for each immune marker: APxAD, APxMS, ADxMS, and APxADxMS. Missingness in covariates or immune markers was handled by listwise exclusion.

### Ethics

Participation in the TOP study required written informed consent. The study was approved by the Regional Committee for Medical and Health Research Ethics (2009/2485) and conducted in accordance with the Declaration of Helsinki.

## Results

### Sample characteristics

The SCZ group was younger than the BD and HC groups (*p* < 0.001), and the BD group had a lower proportion of male participants compared to SCZ and HC (*p* < 0.001). HC had a lower BMI than both patient groups (*p* < 0.001). PANSS total scores were higher in SCZ than BD (*p* < 0.001), and recent illicit substance use was more common in BD than SCZ (*p* = 0.021). Freezer storage time was shorter in HC than in the patient groups (*p* < 0.001). AP use was more frequent in SCZ than BD (*p* < 0.001), whereas AD use and MS use were more frequent in BD than SCZ (*p* = 0.008 and *p* < 0.001, respectively). See Table 1 for additional sample and psychotropic agent descriptives, including differences between groups.

Differences in levels of immune markers were found between SCZ and/or BD and HC for most immune markers (*p* < 0.05), except for BAFF, A2M, GFAP, VCAM-1, PSEL, IL-18RAP, CXCL16, OPG, ALCAM, MPO, vWF, PARK7, and sCD14. Specifically, BD-2, CRP, and sIL-2R levels were higher in both SCZ and BD compared to HC, and CRP levels were higher in SCZ than in BD. See Supplementary Tables 3 and 5 for details.

### Associations between immune marker levels and psychotropic agent use, dose, and serum concentration

**Figure 1.**
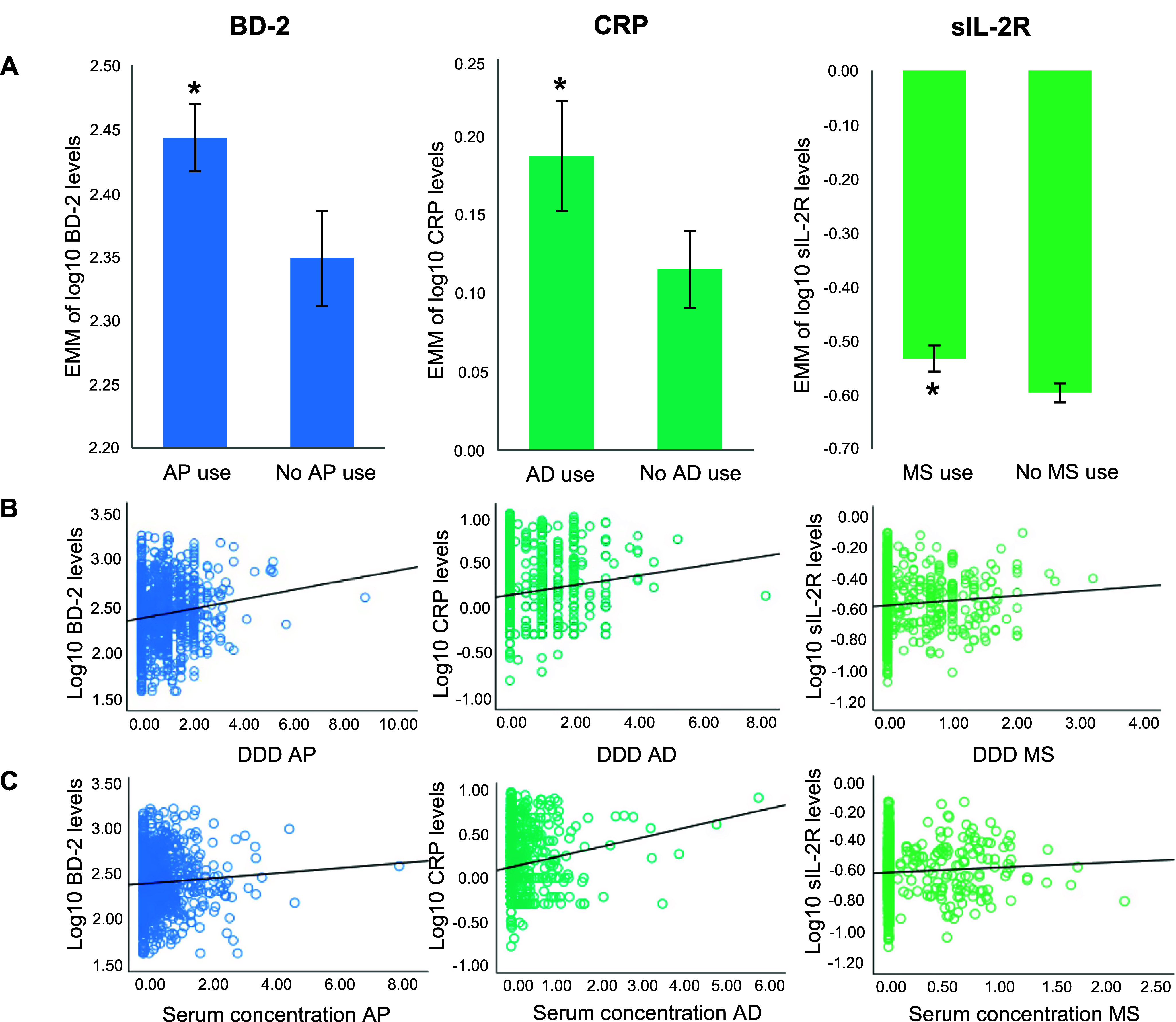
Associations between immune marker levels and psychotropic agent use. (A) Estimated marginal means (EMM) with 95% confidence intervals of log10-transformed immune marker levels by psychotropic agent class (AP, AD, MS), adjusted for age, sex, BMI, diagnosis, PANSS total score, and plasma freezer storage time. (B) Scatter plots showing unadjusted associations between defined daily dose (DDD) and log10-transformed immune marker levels. (C) Scatter plots showing unadjusted associations between serum concentrations and log10-transformed immune marker levels. Asterisk (*) indicates associations significant at *p* < 0.001. Abbreviations: Antidepressant agents (AD), Antipsychotic agents (AP), Defined daily dose (DDD), Estimated marginal means (EMM), Mood stabilizing agents (MS; antiepileptics and lithium).

AP use was associated with higher plasma levels of BD-2 relative to no AP use (*β* = 0.094, *p* = 0.8E-4), AD use with higher serum levels of CRP (*β* = 0.072, *p* = 0.8E-3), and MS use with higher plasma levels of sIL-2R (*β* = 0.063, *p* = 0.9E-4). Table 3 shows effect estimates and *p*-values for various steps of adjustments. In subgroup analyses, sIL-2R was significantly associated with AE use (*β* = 0.061, *p =* 0.5E-3), and nominally with lithium use (*β* = 0.062, *p =* 0.024). Results of the sensitivity analyses showed consistent, though slightly attenuated associations (DDD and serum concentration adjustments, respectively: AP and BD-2, *p* = 0.002 and *p* = 0.005; AD and CRP, *p* = 0.003 and *p* = 0.009; MS and sIL-2R, *p* = 0.004 and *p* = 0.009, Supplementary Table 6). See Figure 1 for associations between immune marker levels and psychotropic agent use.

**Table 3.** Association analyses between psychotropic agent class use and immune markers^a^

Immune marker	Unadjusted			Age + sex			Age + sex + BMI			Age + sex + BMI + diagnosis			Age + sex + BMI + diagnosis + PANSS			Age + sex + BMI + diagnosis+ PANSS + FT		
	AP	AD	MS	AP	AD	MS	AP	AD	MS	AP	AD	MS	AP	AD	MS	AP	AD	MS
BD–2	**0.101** **(0.4E–5)**	0.012 (0.576)	0.032 (0.160)	**0.094** **(0.2E–4)**	0.022 (0.321)	0.039 (0.087)	**0.089** **(0.7E–4)**	0.027 (0.226)	0.043 (0.070)	**0.087** **(0.2E–3)**	0.030 (0.191)	0.070 (0.007)	**0.093** **(0.1E–3)**	0.031 (0.181)	0.074 (0.004)	**0.094** **(0.8E–4)^c^**	0.030 (0.188)	0.075 (0.004)
CRP	**0.091** **(0.1E–3)**	**0.091** **(0.8E–4)**	0.028 (0.240)	**0.096** **(0.5E–4)**	**0.091** **(0.7E–4)**	0.009 (0.697)	**0.076** **(0.6E–3)**	**0.077** **(0.3E–3)**	´–0.017 (0.445)	0.062 (0.009)	**0.082** **(0.2E–3)**	0.007 (0.768)	0.065 (0.006)	**0.080** **(0.2E–3)**	0.008 (0.749)	0.063 (0.008)	**0.072** **(0.8E–3)^c^**	´–0.001 (0.971)
sIL–2R	0.032 (0.029)	0.004 (0.755)	0.041 (0.004)	0.028 (0.051)	0.009 (0.527)	**0.050** **(0.5E–3)**	0.031 (0.039)	0.008 (0.544)	**0.052** **(0.4E–3)**	0.031 (0.052)	0.009 (0.520)	**0.066** **(0.4E–4)**	0.034 (0.032)	0.004 (0.757)	**0.065** **(0.5E–4)**	0.035 (0.030)	0.003 (0.843)	**0.063** **(0.9E–4)** ^b^ ^,^^c^

Abbreviations: AD, antidepressant agents; AE, antiepileptic agents; AP, antipsychotic agents; BD-2, beta defensin 2; BMI, body mass index; CRP, C-reactive protein; FT, freezer storage time; MS, mood stabilizing agents; PANSS, positive and negative syndrome score; sIL-2R, total score, soluble interleukin 2 receptor.

a Linear regression results reported with *β* (*p*-values) at different steps of covariate adjustments.

b Subgroup analyses: sIL-2R and AE use (*β* = 0.061, *p =* 0.5E-3) and lithium use (*β* = 0.062, *p =* 0.024).

c Model statistics: AP use and BD-2 (df = 7, F = 4.808, *p* < 0.001, R^2^ = 0.035), AD use and CRP (df = 7, F = 39.621, *p* < 0.001, R^2^ = 0.207), MS use and sIL-2R (df = 7, F = 5.353, *p* < 0.001, R^2^ = 0.051).

The secondary analyses revealed that DDD AP was positively associated with BD-2 levels (*β* = 0.045, *p* = 2.3E-4), DDD AD was positively associated with CRP levels (*β* = 0.039, *p* = 0.001), and DDD MS was positively associated with sIL-2R levels (*β* = 0.048, *p* = 0.001). Serum concentration of AD was nominally associated with CRP (*β* = 0.072, *p* = 0.002), while serum concentrations of MS and AP were not significantly associated with sIL-2R (*β* = 0.038, *p* = 0.168) and BD-2 (*β* = 0.032, *p* = 0.088), respectively. Subgroup analyses of sIL-2R with serum concentrations of AE and lithium were not significant (*β* = 0.039, *p* = 0.245 and *β* = 0.033, *p* = 0.827, respectively). See Supplementary Table 7 for details.

No other significant associations were found between psychotropic agent classes and immune markers (Supplementary Table 8). Diagnosis-stratified results are presented in Supplementary Table 9.

Exploratory analyses identified an interaction between AD and MS use for ALCAM levels (*β* = −0.056, *p* = 0.001). None of the other tested interactions between psychotropic agent classes were significant across the 44 remaining immune markers.

## Discussion

Our main findings were that use of AP, AD, and MS in individuals with SMDs was associated with elevated levels of BD-2, CRP, and sIL-2R, respectively. These associations were observed both when comparing users versus non-users of psychotropic agents, in relation to dose, and partially in relation to serum concentrations. No other significant associations were found between these psychotropic agent classes and the broad immune marker panel, suggesting minor and specific immunomodulatory associations of psychotropic agent use.

Beta-defensins, including BD-2, have extensive innate immune effects such as antimicrobial activity and immunomodulatory functions [69]. BD-2 is thought to influence central nervous system immune processes through effects on dendritic cells, and reduced BD-2 expression has been associated with neurodegenerative conditions involving immune dysregulation and inflammation [69]. While SMDs are primarily considered to have a neurodevelopmental origin, neurodegenerative features are also indicated [70], potentially linked to positive symptoms [71]. Notably, APs are suggested to have neuroprotective properties, including dampening of microglial activation [72]. Moreover, evidence indicates peripheral immune homeostatic and anti-inflammatory effects of beta-defensins by e.g., cytokine signaling regulation [73]. AP may modulate immune activity through various mechanisms, including interactions with dopamine, serotonin, and acetylcholine receptors expressed on immune cells [74]. However, there is limited research on the association between beta-defensins and SMDs or their treatment, and to the best of our knowledge, this is the first study to demonstrate an association with AP. Our finding is strengthened by the significant associations with dose, and one might speculate that AP use improves regulatory functions of BD-2 including mediating immune homeostatic, anti-inflammatory, and neuroprotective effects. Clinically, such effects could be relevant for symptom control and neuroprotection in subgroups of patients, particularly those with immune dysregulation or treatment resistance [75].

CRP is an acute-phase protein induced by pro-inflammatory cytokines such as IL-1β and IL-6, reflecting non-specific systemic inflammation [76]. Prior studies of AD use and CRP in SCZ and BD are limited. Ding et al. [38] reported reduced CRP levels after 8 weeks of escitalopram add-on treatment in treatment-resistant SCZ, while Edberg et al. [39] reported reductions in CRP between weeks 4 and 8 in BD with treatment-resistant depression, but no change between baseline and week 8. Discrepancies with the current findings may reflect different sample characteristics, such as treatment resistance, and differences in age and duration of illness. A meta-analysis of MDD found no consistent association between AD treatment and CRP [77]. There are, however, some individual studies that support our findings, such as those by Chang et al. [78] and Carboni et al. [79], which found elevated levels of CRP with AD treatment. Associations between AD use and pro-inflammatory markers in MDD mainly include decreased levels of TNF-α and IL-6 following AD treatment [80, 81]. However, pro-inflammatory effects have been suggested through decreased levels of the anti-inflammatory IL-10 [81]. The mechanisms remain unclear, but might involve metabolic disturbances of AD or effects on immune cell serotonin receptors [37, 82, 83]. Importantly, to the best of our knowledge, most single studies in the literature are smaller and not as well-adjusted as the current one. Our findings are further supported by the associations with dose and, suggestively, serum concentration, and one might speculate that pro-inflammatory effects of AD use in SCZ and BD lead to a less favorable clinical course in subgroups of patients. Notably, prior evidence links elevated CRP to poorer clinical outcomes in SMDs [76, 84]. For instance, the proposed mania and rapid cycling inducing effects of AD in BD might be relevant to study in this context, given the inflammatory activity associated with these conditions [85, 86]. The finding supports further investigation of CRP or related markers as potential indicators for monitoring or stratifying patients during AD treatment, and clinicians should keep in mind the potential for subtle negative clinical effects of AD treatment in the SMD population.

Elevated sIL-2R is generally considered to reflect T-cell activation by triggering of IL-2R production and shedding through elevated expression of IL-2 [87]. Several studies have demonstrated altered levels of IL-2 components in SMDs compared to HC [11, 33, 88]. Importantly, sIL-2R may act as a decoy receptor and dampen IL-2 signaling [87, 89], potentially promoting anti-inflammatory effects [87]. Thus, previous findings of elevated sIL-2R in depressive, euthymic, and manic phases of BD, in addition to a positive correlation between sIL-2R and symptom severity in mania [88, 90], might indicate an inflammatory state with enhanced immune activation, but possibly also a pro-homeostatic compensatory mechanism. However, as IL-2 has pleiotropic immune functions, with both stimulatory and suppressive effects [91], the interpretation of these findings remains complex. The limited literature on sIL-2R and psychotropic treatment in SMDs is inconclusive, including reduced sIL-2R levels following lithium treatment [92], a non-significant lower level of sIL-2R after valproate treatment [93], and no clear association between MS use and sIL-2R in a recent review [26]. The current sample is, to the best of our knowledge, by far the largest single sample in which the association between MS use and sIL-2R is examined, and included extensive covariate adjustment. The association was further supported by a correlation with dose and, nominally with serum concentration. While the positive association might indicate an inflammatory effect of MS use, potential anti-inflammatory effects of MS through increased shedding of membrane-bound IL-2R and increased levels of IL-2 decoy receptors are not implausible. Immunosuppressive effects of certain MS have been suggested through inhibition of protein synthesis and suppression of lymphocyte activity [29]. Interestingly, levels of IL-2 have been reported to be reduced in vitro after treatment with MS [28]. The clinical relevance of the current finding is unclear, but one might speculate that an immune homeostatic process may be contributing to the mood-stabilizing effect of MS.

The study has several strengths, including a large, well-characterized sample, a broad immune marker panel, and detailed psychotropic medication exposure data, allowing investigation of associations between psychotropic agent use and immune markers in SMDs with extensive adjustments. The immune markers reflect relevant pathways, although sgp130 and sTNF-R1 are less investigated components of the IL-6 [94] and TNF pathways [95]. Moreover, the selection includes markers linked with neuroinflammation, such as SERPINA3, BAFF, and YKL-40; however, peripheral measurements clearly limit the interpretation relative to central processes. Nevertheless, future studies could benefit from investigating broader panels linked to neuroinflammation and microglial activation. Information on psychotropic dose and serum concentrations enabled us to capture various aspects of exposure-immune associations, supporting the indicated associations with use. However, the study’s cross-sectional, non-randomized design limits causal inference; the larger TOP study also includes follow-up examinations of patients at varying intervals of years, but is not designed for rigorous longitudinal analysis of medication effects. Thus, although no follow-up is currently planned, longitudinal studies are needed to clarify temporal and causal relationships. Particularly, reverse causality cannot be excluded, despite the demonstrated associations across use, dose, and serum concentration; i.e., immune abnormalities may have influenced clinical presentation and thereby affected treatment decisions. Moreover, the lack of longitudinal data hinders examinations of associations related to changes in illness severity. Lastly, The sensitivity analyses showed attenuations in significance levels for all identified associations; however, there were relatively minor reductions in effect sizes. As the sensitivity analyses involved more than a doubling of number of covariates and some loss of participants due to missingness in covariate data, the results seem robust, and attenuations are likely related to the size and complexity as well as sample reductions. Nevertheless, the significant findings should be interpreted with caution given the large proportion of negative results.

In the current study, we demonstrated associations between elevated levels of BD-2, CRP, and sIL-2R and AP, AD, and MS use, respectively, in a large and well-characterized sample of individuals with SMDs. These findings, supported by associations dose, and, in part, serum concentrations, underscore the need to account for medication effects when interpreting immune alterations in SMDs. The associations suggest that psychotropic agents may modestly influence specific immune pathways of relevance for immune homeostasis and inflammatory regulation in SMDs. Importantly, our findings support the translational potential of immune-informed stratification in SMDs, aligning with emerging evidence that inflammatory biomarkers can help identify subgroups with differential treatment response [96–99]. Nevertheless, the lack of associations for most immune markers indicates that psychotropic agents exert limited effects on broader immune signaling, and further longitudinal studies are warranted to clarify directionality and clinical implications.

## Supporting information

10.1192/j.eurpsy.2025.10104.sm001 Ormerod et al. supplementary material 1 Ormerod et al. supplementary material

10.1192/j.eurpsy.2025.10104.sm002 Ormerod et al. supplementary material 2 Ormerod et al. supplementary material

10.1192/j.eurpsy.2025.10104.sm003 Ormerod et al. supplementary material 3 Ormerod et al. supplementary material

## Data Availability

Due to ethical restrictions and the need to protect participant anonymity, the datasets generated and/or analyzed during the present study are not publicly available.

## Supplementary material

The supplementary material for this article can be found at http://doi.org/10.1192/j.eurpsy.2025.10104.
