# Supplementary material for: Minor immunomodulatory effects of psychotropics suggested in severe mental disorders: Associations of antipsychotics with beta defensin 2, antidepressants with C-reactive protein, and mood stabilizers with soluble interleukin 2 receptor
Source: Eur Psychiatry. 2025 Sep 12;68(1):e140. doi: 10.1192/j.eurpsy.2025.10104 (PMC12538189; doi:10.1192/j.eurpsy.2025.10104)
Supplement: Ormerod et al. supplementary material 2 — Ormerod et al. supplementary material [file S0924933825101041sup002.docx]

**Supplementary material**

**Content**

- **Supplementary Table 1** Psychotropic agent use by patients
- **Supplementary Table 2** Somatic agent use by patients
- **Supplementary Table 3** Immune marker levels per group
- **Supplementary Table 4** Removal of data points based on residual distributions
- **Supplementary Table 5** Immune marker levels of matched groups
- **Supplementary Table 6** Sensitivity analysis of associations between psychotropic agent class use and immune markers
- **Supplementary Table 7** Association analyses between immune markers and psychotropic dose (DDD) and serum concentrations
- **Supplementary Table 8** Association analyses between psychotropic agent class use and immune markers
- **Supplementary Table 9** Association analyses between psychotropic agent class use and immune markers in subdiagnoses

**Supplementary Table 1** Psychotropic agent use by patients

See Excel file *" Supplementary Table 1 "*.

| **Supplementary Table 2** Somatic agent use by patients | | |
| --- | --- | --- |
| **Somatic agents** | **SCZ (N = 777)**  **N (%)** | **BD (N = 438)**  **N (%)** |
| Anti-inflammatory/immunomodulatory agents | 16 (2.1) | 6 (1.4) |
| Antidiabetic agents | 8 (1.0) | 5 (1.1) |
| Cardiovascular/lipid modifying agents | 26 (3.3) | 17 (3.9) |
| Gastrointestinal agents | 18 (2.3) | 6 (1.4) |
| Other^a^ | 86 (11.1) | 82 (18.7) |
| ^a^Other: vitamins, minerals, endocrine agents, antimigraine agents, analgetic agents, haematological agents, mucolytic agents.  Abbreviations: Bipolar disorders (BD), Schizophrenia spectrum disorders (SCZ). | | |

| **Supplementary Table 3** Immune marker levels per group^a^ | | | | | | |
| --- | --- | --- | --- | --- | --- | --- |
| **Immune markers** | **SCZ**  **(N = 777)**  **Median (IQR)** | **BD**  **(N = 438)**  **Median (IQR)** | **HC**  **(N = 1008)**  **Median (IQR)** | ***p*-value^b^** | **Pairwise comparisons^b^** |  |
| IL1-RA (pg/mL) | 240 (335) | 205 (320) | 196 (267) | **<0.001** | HC, BD<SCZ |  |
| IL-18 (pg/mL) | 1000 (1554) | 774 (1352) | 699 (1156) | **<0.001** | HC<SCZ; SCZ<BD |  |
| IL-18BP (ng/mL) | 6.36 (2.99) | 5.85 (2.45) | 5.48 (2.22) | **<0.001** | HC<SCZ, BD; BD<SCZ |  |
| IL-18RAP (pg/mL) | 47.7 (19.6) | 46.3 (17.1) | 48.6 (20.1) | 0.15 | - |  |
| IL-18R1 (ng/mL) | 0.86 (0.46) | 0.81 (0.41) | 0.79 (0.38) | **<0.001** | HC, BD<SCZ |  |
| sTNF-R1 (ng/mL) | 1.76 (0.73) | 1.68 (0.57) | 1.60 (0.77) | **<0.001** | HC<SCZ, BD |  |
| BAFF (pg/mL) | 245 (156) | 238 (171) | 238 (156) | 0.54 | - |  |
| OPG (ng/mL) | 1.28 (0.47) | 1.36 (0.55) | 1.32 (0.45) | 0.06 | - |  |
| APRIL (pg/mL) | 259 (238) | 262 (243) | 334 (279) | **<0.001** | BD, SCZ<HC |  |
| sgp130 (ng/mL) | 217 (57.8) | 210 (57.4) | 223 (57.9) | **0.002** | BD<HC |  |
| GROα (pg/mL) | 21.2 (17.6) | 20.9 (16.3) | 22.6 (17.0) | **0.015** | BD, SCZ<HC |  |
| SDF1α (pg/mL) | 1481 (849) | 1519 (850) | 1604 (935) | **0.010** | SCZ<HC |  |
| Eotaxin (pg/mL) | 2.40 (3.87) | 2.66 (3.92) | 3.11 (5.50) | **<0.001** | SCZ, BD<HC |  |
| RANTES (ng/mL) | 79.7 (72.3) | 75.6 (73.6) | 91.1 (77.9) | **<0.001** | SCZ, BD<HC |  |
| CXCL16 (ng/mL) | 15.7 (8.60) | 15.4 (7.63) | 15.2 (8.62) | 0.13 | - |  |
| MAdCAM-1 (ng/mL) | 7.65 (4.29) | 7.43 (4.05) | 6.98 (3.58) | **<0.001** | HC<SCZ, BD |  |
| JAMA (ng/mL) | 1.32 (0.79) | 1.28 (0.74) | 1.19 (0.74) | **<0.001** | HC<SCZ, BD |  |
| NCAD (ng/mL) | 6.99 (3.29) | 6.99 (2.83) | 6.76 (2.83) | **0.048** | HC<SCZ |  |
| ICAM-1 (ng/mL) | 275 (117) | 269 (106) | 250 (97.0) | **<0.001** | HC<SCZ, BD |  |
| VCAM-1 (ng/mL) | 485 (171) | 486 (167) | 497 (155) | 0.57 | - |  |
| PSEL (ng/mL) | 42.7 (31.2) | 41.1 (34.8) | 45.0 (33.5) | 0.29 | - |  |
| ALCAM (ng/mL) | 38.0 (12.1) | 37.9 (12.3) | 38.9 (10.34) | 0.09 | - |  |
| HNP1-3 (ng/mL) | 7.61 (5.33) | 7.19 (5.74) | 8.00 (6.51) | **0.040** | BD<HC |  |
| BD-1 (ng/mL) | 11.8 (4.45) | 13.0 (6.02) | 12.2 (4.84) | **<0.001** | SCZ, HC<BD, HC |  |
| BD-2 (ng/mL) | 268 (283) | 250 (244) | 206 (224) | **<0.001** | HC<SCZ, BD |  |
| sIL-2R (ng/mL) | 0.28 (0.18) | 0.25 (0.16) | 0.25 (0.14) | **<0.001** | HC<SCZ, BD |  |
| MIF (ng/mL) | 24.8 (46.5) | 22.3 (38.8) | 20.6 (34.5) | **0.019** | HC<SCZ |  |
| sCD14 (ng/mL) | 1909 (459) | 1954 (422) | 1959 (476) | 0.053 | - |  |
| S100B (ng/mL) | 107 (40.1) | 101 (26.2) | 107 (33.1) | **0.002** | BD<SCZ, HC |  |
| Furin (ng/mL) | 0.40 (0.26) | 0.36 (0.24) | 0.38 (0.27) | **0.005** | BD<SCZ, HC |  |
| GFAP (pg/mL) | 148 (122) | 149 (130) | 152 (140) | 0.87 | - |  |
| NSE (ng/mL) | 2.42 (3.76) | 2.67 (3.82) | 3.10 (5.28) | **<0.001** | SCZ, BD<HC |  |
| A2M (µg/mL) | 13.6 (10.5) | 14.5 (10.6) | 13.8 (9.13) | 0.22 | - |  |
| CRP (µg/mL) | 1.28 (2.10) | 1.02 (2.10) | 1.00 (1.20) | **<0.001** | HC<SCZ, BD; BD<SCZ |  |
| CAT-S (ng/mL) | 5.08 (2.56) | 4.86 (2.04) | 4.63 (1.89) | **<0.001** | HC<SCZ |  |
| Gal-3 (ng/mL) | 2.87 (3.63) | 2.52 (3.28) | 3.14 (3.62) | **0.005** | BD<SCZ, HC |  |
| SERPINA3 (µg/mL) | 1.25 (0.88) | 1.34 (0.93) | 1.24 (0.83) | **0.015** | HC, SCZ<BD |  |
| YKL-40 (ng/mL) | 37.0 (28.9) | 39.6 (31.3) | 32.4 (19.9) | **<0.001** | HC<SCZ, BD |  |
| MPO (ng/mL) | 264 (418) | 254 (559) | 257 (500) | 0.92 | - |  |
| PARK7 (ng/mL) | 4.50 (2.66) | 4.16 (2.37) | 4.49 (3.17) | 0.06 | - |  |
| PTX3 (ng/mL) | 2.81 (2.15) | 2.64 (1.90) | 2.91 (2.19) | **0.007** | BD<HC |  |
| vWF (AU) | 76.3 (89.5) | 66.4 (71.2) | 69.2 (74.9) | 0.051 | - |  |
| IGFBP4 (ng/mL) | 157 (51.0) | 159 (56.7) | 144 (46.8) | **<0.001** | HC<SCZ, BD |  |
| BDNF (ng/mL) | 4.99 (5.30) | 4.88 (4.32) | 6.10 (4.95) | **<0.001** | SCZ, BD<HC |  |
| DKK1 (ng/mL) | 0.76 (0.92) | 0.64 (0.65) | 0.83 (0.99) | **<0.001** | BD<SCZ, HC |  |
| ^a^Reported elsewhere [1-8].  ^b^Kruskal-Wallis test and Mann-Whitney *U-*test  Available immune data in total sample, N (%): CXCL16: 635 (44.3); MIF 640 (44.7); sTNF-R1, ALCAM, sgp130, MPO, YKL-40, vWF, BDNF, CAT-S: 642 (44.8); OPG, IL-1RA, sIL-2R, PARK7, PTX3, IGBP4, DKK1, Gal-3: 643 (44.9); IL-18: 1151 (80.4); IL-18R1: 1166 (81.4); MAdCAM-1: 1197 (83.6); S100B, Furin, GFAP, IL-18RAP: 1199 (83.7); A2M, SERPINA3, NSE, VCAM-1, PSEL, IL-18BP, BD-1: 1203 (84.0); Eotaxin, HNP1-3: 1204 (84.1); BAFF, APRIL, GROα, SDF1α, RANTES, JAMA, NCAD, ICAM-1, BD-2: 1206 (84.2); CRP 1349 (94.2).  Abbreviations: Bipolar disorder (BD), Healthy controls (HC), Interquartile range (IQR), Schizophrenia spectrum disorder (SCZ). | | | | | | |

| **Supplementary Table 4** Removal of data points based on residual distributions^a^ | |
| --- | --- |
| 3 x interquartile range, percent removal in parenthesis | sTNF-R1 (1.3), CXCL16 (2.6), OPG (2.1), ALCAM (0.9), sgp130 (0.3), YKL-40 (2.3), JAMA (1.7), VCAM-1 (0.9), IL-18BPa (4.3), IL-18R1 (0.5), BD-1 (4.8) |
| 1.5 x interquartile range, percent removal in parenthesis | IL-1RA (2.8), sIL-2R (6.6), vWF (0.5), BDNF (0.7), CAT-S (2.5), PARK7 (5.9), PTX3 (4.2), IGFBP4 (3.8), sCD14 (1.5), DKK1 (2.9), Gal-3 (1.9), MIF (4.3), CRP 0.1), BAFF (9.0), APRIL (5.0), S100B (12.4), GFAP (12.1), Furin (3.4), NSE (0.6), A2M (1.0), SA3 (2.0), GROα (2.2), SDF1α (5.1), Eotaxin (1.5), RANTES (0.6), MadCAM-1 (8.3), NCAD (16.0), ICAM-1 (3.1), IL-18RAP (21.3), HNP1-3 (15.2), BD-2 (2.6) |
| ^a^To comply with the normality assumption of linear regressions, the immune marker data were log-transformed and data points iteratively removed based on residuals exceeding 3 x or 1.5 x interquartile range (IQR) below or above the lower and upper quartile, respectively. | |

| **Supplementary Table 5** Immune marker levels of matched groups^a^ | | | | |
| --- | --- | --- | --- | --- |
|  | **Matched groups^b^** | | **Matched groups^c^** | |
| **Immune markers** | **SCZ**  **Median (IQR)** | **HC**  **Median (IQR)** | **BD**  **Median (IQR)** | **HC**  **Median (IQR)** |
| IL1-RA (pg/mL) | 228 (309) | 183 (248) | 204 (309) | 183 (248) |
| IL-18 (pg/mL) | 915 (1618) | 516 (977) | 581 (1661) | 516 (977) |
| IL-18BP (ng/mL) | 6.42 (3.18) | 5.54 (2.35) | 6.05 (2.73) | 5.54 (2.35) |
| IL-18RAP (pg/mL) | 47.3 (19.9) | 47.3 (14.1) | 47.5 (19.1) | 47.3 (14.1) |
| IL-18R1 (ng/mL) | 0.87 (0.48) | 0.80 (0.43) | 0.79 (0.47) | 0.80 (0.43) |
| sTNF-R1 (ng/mL) | 1.75 (0.74) | 1.58 (0.62) | 1.67 (0.60) | 1.58 (0.62) |
| BAFF (pg/mL) | 267 (161) | 250 (158) | 258 (173) | 250 (158) |
| OPG (ng/mL) | 1.24 (0.45) | 1.31 (0.44) | 1.30 (0.53) | 1.31 (0.44) |
| APRIL (pg/mL) | 250 (236) | 301 (263) | 226 (198) | 301 (263) |
| sgp130 (ng/mL) | 213 (55.7) | 220 (57.0) | 202 (53.3) | 220 (57.0) |
| GROα (pg/mL) | 23.1 (19.3) | 24.3 (17.6) | 24.3 (18.1) | 24.3 (17.6) |
| SDF1α (pg/mL) | 1544 (827) | 1594 (864) | 1636 (802) | 1594 (864) |
| Eotaxin (pg/mL) | 132 (97.9) | 128 (87.3) | 129 (80.9) | 128 (87.3) |
| RANTES (ng/mL) | 87.3 (79.2) | 94.1 (79.5) | 89.5 (77.8) | 94.1 (79.5) |
| CXCL16 (ng/mL) | 15.3 (8.65) | 15.2 (8.82) | 15.4 (7.71) | 15.2 (8.82) |
| MAdCAM-1 (ng/mL) | 7.60 (4.35) | 7.13 (3.07) | 7.30 (3.89) | 7.13 (3.07) |
| JAMA (ng/mL) | 1.31 (0.80) | 1.17 (0.64) | 1.27 (0.79) | 1.17 (0.64) |
| NCAD (ng/mL) | 7.29 (3.16) | 6.77 (3.59) | 7.06 (2.99) | 6.77 (3.59) |
| ICAM-1 (ng/mL) | 275 (121) | 248 (86.4) | 265 (105) | 248 (86.4) |
| VCAM-1 (ng/mL) | 478 (183) | 477 (149) | 480 (163) | 477 (149) |
| PSEL (ng/mL) | 46.6 (33.9) | 46.8 (41.6) | 47.9 (41.1) | 46.8 (41.6) |
| ALCAM (ng/mL) | 35.9 (12.8) | 40.3 (10.8) | 36.8 (11.2) | 40.3 (10.8) |
| HNP1-3 (ng/mL) | 8.16 (6.38) | 9.13 (9.46) | 7.67 (8.18) | 9.13 (9.46) |
| BD-1 (ng/mL) | 11.5 (4.56) | 11.4 (4.55) | 12.3 (5.50) | 11.4 (4.55) |
| BD-2 (ng/mL) | 271 (287) | 178 (169) | 264 (258) | 178 (169) |
| sIL-2R (ng/mL) | 0.28 (0.18) | 0.25 (0.17) | 0.25 (0.16) | 0.25 (0.17) |
| MIF (ng/mL) | 20.9 (33.1) | 23.2 (33.7) | 20.7 (34.5) | 23.2 (33.7) |
| sCD14 (ng/mL) | 1875 (491) | 1939 (500) | 1930 (463) | 1939 (500) |
| S100B (ng/mL) | 107 (45.3) | 108 (34.9) | 102 (24.0) | 108 (34.9) |
| Furin (ng/mL) | 0.43 (0.29) | 0.43 (0.32) | 0.39 (0.26) | 0.43 (0.32) |
| GFAP (pg/mL) | 155 (159) | 154 (123) | 149 (120) | 154 (123) |
| NSE (ng/mL) | 2.43 (4.33) | 3.01 (5.37) | 2.63 (4.89) | 3.01 (5.37) |
| A2M (µg/mL) | 13.5 (10.5) | 13.8 (9.98) | 14.5 (11.4) | 13.8 (9.98) |
| CRP (µg/mL) | 1.20 (2.10) | 1.00 (1.50) | 1.00 (2.20) | 1.00 (1.50) |
| CAT-S (ng/mL) | 4.81 (2.26) | 4.63 (2.06) | 4.54 (2.05) | 4.63 (2.06) |
| Gal-3 (ng/mL) | 3.03 (3.79) | 3.40 (3.31) | 2.79 (3.32) | 3.40 (3.31) |
| SERPINA3 (µg/mL) | 1.24 (0.87) | 1.22 (0.92) | 1.33 (0.91) | 1.22 (0.92) |
| YKL-40 (ng/mL) | 35.0 (24.2) | 34.3 (24.2) | 36.1 (27.2) | 34.3 (24.2) |
| MPO (ng/mL) | 219 (392) | 268 (472) | 214 (489) | 268 (472) |
| PARK7 (ng/mL) | 4.27 (2.42) | 4.25 (3.09) | 4.13 (2.40) | 4.25 (3.09) |
| PTX3 (ng/mL) | 2.58 (2.17) | 3.27 (2.58) | 2.54 (1.88) | 3.27 (2.58) |
| vWF (AU) | 74.0 (86.7) | 72.2 (72.6) | 66.2 (68.3) | 72.2 (72.6) |
| IGFBP4 (ng/mL) | 157 (47.9) | 150 (51.9) | 156 (46.5) | 150 (51.9) |
| BDNF (ng/mL) | 4.55 (4.75) | 6.15 (5.80) | 4.66 (4.02) | 6.15 (5.80) |
| DKK1 (ng/mL) | 0.75 (0.96) | 0.81 (1.20) | 0.65 (0.73) | 0.81 (1.20) |
| ^a^Matching on age, sex, and BMI using propensity score matching (R package Matchit_4.5.5).  ^b^SCZ and HC matched groups (N = 728, 59.9% males, both groups) [median (IQR)]: SCZ, age 27.0 (14.0), BMI 25.1 (6.6); HC, age 30.0 (12.0), BMI 24.6 (4.78).  ^c^BD and HC matched groups (N = 421, 40.4% males, both groups) [median (IQR)]: BD, age 31.0 (17.0), BMI 25.4 (5.5); HC, age 32.0 (15.0), BMI 25.1 (4.24).  Abbreviations: Bipolar disorder (BD), Healthy controls (HC), Interquartile range (IQR), Schizophrenia spectrum disorder (SCZ). | | | | |

| **Supplementary Table 6** Sensitivity analyses of associations between psychotropic agent class use and immune markers^a^ | | | |
| --- | --- | --- | --- |
| **Immune marker** | **AP** | **AD** | **MS** |
| BD-2^b^ | 0.086 (0.002) |  |  |
| CRP^b^ |  | 0.070 (0.003) |  |
| sIL-2R^b^ |  |  | 0.052 (0.004) |
| BD-2^c^ | 0.084 (0.005) |  |  |
| CRP^c^ |  | 0.067 (0.009) |  |
| sIL-2R^c^ |  |  | 0.050 (0.009) |
| ^a^Linear regressions results reported with *β* (*p*-value).  ^b^Model: Adjustments for age, sex, BMI, diagnosis, PANSS total score, freezer storage time, CRP (except for the CRP analysis), smoking, education, duration of illness, illicit substance use (past two weeks), time of blood sampling, DDD AP, DDD AD and DDD MS (DDD of the medication categories not specifically tested).  ^c^Model: Adjustments for age, sex, BMI, diagnosis, PANSS total score, freezer storage time, CRP (except for the CRP analysis), smoking, education, duration of illness, illicit substance use (past two weeks), time of blood sampling, serum concentration of AP, AD and MS (serum concentration of the medication classes not specifically tested).  Percentage of participants lost from main analyses to sensitivity analyses due to missing covariate data: ^b^BD-2 6.8 %, CRP 1.5 %, sIL-2R 6.4 %; ^c^BD-2 25.6 %, CRP 25.3 %, sIL-2R 15.9 %. Abbreviations: Antidepressant agents (AD), Antipsychotic agents (AP), Beta defensin 2 (BD-2), Body mass index (BMI), C-reactive protein (CRP), Defined daily dose (DDD), Mood stabilizing agents (MS, antiepileptics and lithium), Positive and Negative Syndrome Scale (PANSS), Soluble interleukin 2 receptor (sIL-2R). | | | |

| **Supplementary Table 7** Association analyses between immune markers and psychotropic dose (DDD) and serum concentrations^a^ | | | |
| --- | --- | --- | --- |
| **Immune marker** | **DDD AP** | **DDD AD** | **DDD MS^b^** |
| BD-2 | 0.045 (2.3E-4) |  |  |
| CRP |  | 0.039 (0.001) |  |
| sIL-2R |  |  | 0.048 (0.001) |
|  | **Serum concentration AP** | **Serum concentration AD** | **Serum concentration MS^c^** |
| BD-2 | 0.032 (0.088) |  |  |
| CRP |  | 0.072 (0.002) |  |
| sIL-2R |  |  | 0.038 (0.168) |
| ^a^Linear regressions reported with *β* (*p*-value). Model: Adjustments for age, sex, BMI, diagnosis, PANSS total score, freezer storage time, and time of blood sampling for serum concentrations.  ^b^Subgroup analyses of MS DDD: AE DDD and sIL-2R (*β* = 0.048, *p* = 0.011), lithium DDD and sIL-2R (*β* = 0.038, *p* = 0.078).  ^c^Subgroup analyses of MS serum concentrations: AE and sIL-2R (*β* = 0.039, *p* = 0.245), lithium and sIL-2R (*β* = 0.033, *p* = 0.827).  Abbreviations: Antidepressant agents (AD), Antiepileptic agents (AE), Antipsychotic agents (AP), Beta defensin 2 (BD-2), Body mass index (BMI), C-reactive protein (CRP), Defined Daily Dose (DDD), Mood stabilizing agents (MS, antiepileptics and lithium), Positive and Negative Syndrome Scale (PANSS), Soluble interleukin 2 receptor (sIL-2R). | | | |

**Supplementary Table 8** Association analyses between psychotropic agent class use and immune markers

See Excel file *" Supplementary Table 8 "*.

| **Supplementary Table 9** Association analyses between psychotropic agent class use and immune markers in subdiagnoses^a^ | | | | | | | | | | |
| --- | --- | --- | --- | --- | --- | --- | --- | --- | --- | --- |
| **Schizophrenia and schizoaffective disorders** | | | | **Bipolar I disorder** | | | **Bipolar II disorder** | | | |
|  | **AP use** | **AD use** | **MS use** | **AP use** | **AD use** | **MS use** | **AP use** | **AD use** | **MS use** |  |
| BD-2 | 0.120 (0.015) | 0.020 (0.621) | 0.119 (0.028) | 0.059 (0.181) | 0.042 (0.363) | 0.042 (0.349) | 0.182 (0.005) | 0.012 (0.838) | 0.042 (0.479) |  |
| CRP | 0.031 (0.542) | 0.098 (0.012) | -0.013 (0.812) | 0.101 (0.016) | 0.104 (0.019) | -0.006 (0.891) | 0.065 (0.371) | 0.109 (0.076) | 0.088 (0.156) |  |
| sIL-2R | 0.075 (0.018) | 0.022 (0.306) | 0.114 (**0.2E-4**) | 0.015 (0.605) | -0.056 (0.051) | 0.008 (0.781) | -0.004 (0.925) | -0.001 (0.988) | 0.063 (0.102) |  |
| ^a^Linear regression results reported with β (p-values) with covariate adjustments including age, sex, BMI, PANSS and freezer storage time.  ^b^Diagnosis group descriptives: Schizophrenia and schizoaffective disorders, N = 554, Bipolar I disorder, N = 271, Bipolar II disorder, N = 143.  ^c^Psychotropic agent class use across diagnoses, N (%): SCZ: AP use = 483 (87.2), AD use = 164 (29.6), MS use = 88 (15.9); Bipolar I disorder: AP use = 167 (61.6), AD use = 87 (32.1), MS use = 161 (59.4); Bipolar II disorder: AP use = 38 (26.6), AD use = 60 (42.0), MS use = 61 (42.7).  Abbreviations: Antidepressant agents (AD), Antiepileptic agents (AE,) Antipsychotic agents (AP), Beta defensin 2 (BD-2), Body mass index (BMI), C-reactive protein (CRP), Freezer storage time (FT), Mood stabilizing agents (MS), Positive and negative syndrome score (PANSS) total score, soluble interleukin 2 receptor (sIL-2R). | | | | | | | | | | |

**References**

1. Andreou D, Steen NE, Jørgensen KN, Smelror RE, Wedervang-Resell K, Nerland S, et al. Lower circulating neuron-specific enolase concentrations in adults and adolescents with severe mental illness. Psychol Med. 2021:1-10. <https://doi.org/10.1017/S0033291721003056>.

2. Hjell G, Szabo A, Mørch-Johnsen L, Holst R, Tesli N, Bell C, et al. Interleukin-18 signaling system links to agitation in severe mental disorders. Psychoneuroendocrinology. 2022;140:105721. <https://doi.org/10.1016/j.psyneuen.2022.105721>.

3. Ormerod MBEG, Ueland T, Werner MCF, Hjell G, Rodevand L, Saether LS, et al. Composite immune marker scores associated with severe mental disorders and illness course. Brain Behav Immun Health. 2022;24:100483. <https://doi.org/10.1016/j.bbih.2022.100483>.

4. Morch RH, Dieset I, Faerden A, Hope S, Aas M, Nerhus M, et al. Inflammatory evidence for the psychosis continuum model. Psychoneuroendocrinology. 2016;67:189-97. <https://doi.org/10.1016/j.psyneuen.2016.02.011>.

5. Engh JA, Ueland T, Agartz I, Andreou D, Aukrust P, Boye B, et al. Plasma Levels of the Cytokines B Cell-Activating Factor (BAFF) and A Proliferation-Inducing Ligand (APRIL) in Schizophrenia, Bipolar, and Major Depressive Disorder: A Cross Sectional, Multisite Study. Schizophr Bull. 2021. <https://doi.org/10.1093/schbul/sbab106>.

6. Sheikh MA, O`Connell KS, Lekva T, Szabo A, Akkouh IA, Osete JR, et al. Systemic cell-adhesion molecules (CAM) in severe mental illness-potential role of intracellular CAM-1 in linking peripheral and neuro-inflammation. Biol Psychiatry. 2022.

7. Szabo A, O'Connell KS, Ueland T, Sheikh MA, Agartz I, Andreou D, et al. Increased circulating IL-18 levels in severe mental disorders indicate systemic inflammasome activation. Brain Behav Immun. 2022;99:299-306. <https://doi.org/10.1016/j.bbi.2021.10.017>.

8. Sæther LS, Ueland T, Haatveit B, Maglanoc LA, Szabo A, Djurovic S, et al. Inflammation and cognition in severe mental illness: patterns of covariation and subgroups. Mol Psychiatry. 2023;28(3):1284-92. <https://doi.org/10.1038/s41380-022-01924-w>.
